# Synthesis of Dimethyl-Substituted Polyviologen and Control of Charge Transport in Electrodes for High-Resolution Electrochromic Displays

**DOI:** 10.3390/polym9030086

**Published:** 2017-03-03

**Authors:** Kan Sato, Ryusuke Mizukami, Takahiro Mizuma, Hiroyuki Nishide, Kenichi Oyaizu

**Affiliations:** Department of Applied Chemistry, Waseda University, Tokyo 169-8555, Japan; satokan@toki.waseda.jp (K.S.); tdwu_300wind@ruri.waseda.jp (R.M.); t.mizuma@fuji.waseda.jp (T.M.)

**Keywords:** electroactive polymer, electrochromic cells, energy storage, electronics

## Abstract

Electrochromic (EC) polymers such as polyviologens have been attracting considerable attention as wet-processable electrodes for EC displays, thanks to their brilliant color change accompanied with reversible redox reactions. To establish wider usage, achieving multicolor and high-resolution characteristics is indispensable. In this paper, we demonstrated that the introduction of substituents such as methyl groups into bipyridine units changed the stereostructure of the cation radicals, and thus shifted the color (e.g., ordinary purple to blue). Also, by relaxing excessive π-stacking between the viologen moieties, the response rate was improved by a factor of more than 10. The controlled charge transport throughout the polyviologen layer gave rise to the fabrication of EC displays which are potentially suitable for the thin film transistor (TFT) substrate as the counter electrodes with submillimeter pixels. The findings can be versatilely used for the new design of polyviologens with enhanced electrochemical properties and high-resolution, multicolor EC displays.

## 1. Introduction

Redox-active polymers have been attracting considerable attention as the key components of various energy-related devices such as charge storage devices, displays, memories, and solar cells due to their splendid electrochemical characteristics [1,2,3,4,5,6,7,8,9]. In particular, electrochromic (EC) polymers represented by polyviologens, polythiophenes, and metallopolymers show high contrast accompanied with reversible redox reactions [3,9,10,11,12,13,14,15,16,17,18]. The polymers were studied as the candidates for wet-processable [19] materials for displays and smart windows. The EC materials usually retain their coloring states even under the open circuit condition, enabling the low power consumption characteristics (i.e., memory effect), which is a significant contrast to liquid crystal and organic light-emitting diode displays [10,11,20]. The absorption wavelength was tuned by introducing substituent to redox-active units, changing conjugation length, and substitution of heteroatoms or metals in case of polythiophenes and metallopolymers [11,12,13,14,15]. Such color tunability and the potentially high reflectance of the EC cells (>70%) [21,22] could be regarded as one of the important advantages against conventional electronic papers (e-papers) [23,24,25]. Still, the color of the polyviologens has been limited to typically violet whilst tuning of chemical structures was common with the low molecular weight compounds [11,26,27,28,29,30,31].

**Figure 1:**
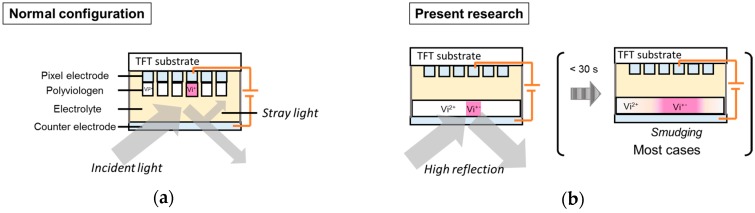
Schematics for electrochromic displays installed with thin film transistor (TFT) substrates and smudging phenomenon. Vi^2+^ and Vi^+^ stands for dication and cation states of viologen units, respectively.

Fabrication of high-resolution, multicolor displays by cost-effective processes is one of the major challenges left in EC technologies. Unlike organic light-emitting diodes and e-papers, the full-color, high pixel EC displays have been hardly ever achieved [21,22,23,32,33]. An active matrix type EC display using polythiophene derivative printed on a thin film transistor (TFT) substrate was recently reported (typical configuration shown in Figure 1a) [33]. However, forming EC materials only on each pixel electrode of a TFT substrate requires fine printing or complicated etching processes which are cost-ineffective. The configuration may not be even appropriate for preparing small pixels of EC electrodes (e.g., pixel size was centimeter scale in the reported device [33]).

As a significant alternative to the conventional configuration of EC displays, a new simple structure with an EC layer formed on the opposite side of the TFT substrate has been recently proposed (Figure 1b) [21,22]. The configuration gave remarkable advantages over conventional one: the highest reflectance of light beyond 70% was achieved for electrochromic cells due to the smaller loss of stray light in the device. Sub-millimeter pixels were easily realized without etching the EC materials. Further, a full color display was also fabricated by using a tandem EC layers. However, the most serious problem of the configuration is that unexpected pixel smudging tends to occur in the region where no voltage is applied. The smudging seems to be caused by charge transport through the EC materials to the open circuit part, but the reason for bleeding and the countermeasure have not been revealed yet, which has prevented the wide commercialization of the EC displays.

**Scheme 1:**
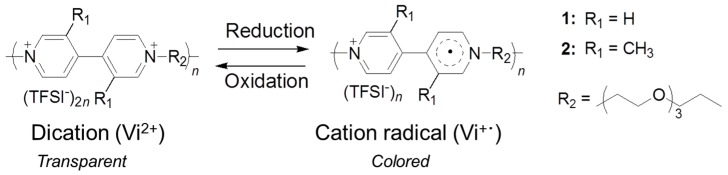
Synthesis of polyviologen **1** and **2**.

In this paper, we firstly synthesized polyviologen with methyl groups introduced in bipyridine rings (Scheme 1). The introduction of methyl groups shifted the color of the polymer from purple to blue due to the conformation change of the redox units. Further, tuning of π-stacking between bipyridine moieties increased the response rate by a factor of >10. A series of electrochemical analysis also clarified that the smudging in Figure 1b was caused by the charge transfer via ITO substrate, and use of alkylbenzene compounds was effective for the suppression. These findings will contribute to the realization of high resolution and full color EC displays which are wet-processable.

## 2. Materials and Methods

### 2.1. Materials

4-Cyano-4′-pentylbiphenyl (5CB), lithium bis(trifluoromethanesulfonyl)imide (LiTFSI), gamma-Butyrolactone (GBL), and lithium trifluoromethanesulfonate (LiCF_3_SO_3_) were purchased from Tokyo Kasei Co., Tokyo, Japan. Tetraethylene glycol di(*p*-toluenesulfonate) was obtained from Sigma-Aldrich Japan, Tokyo, Japan. 1-Ethyl-1-methylpyrrolidinium tetracyanoborate (EMIM TCB) was purchased from Merck Japan, Tokyo, Japan. Other compounds were obtained from Tokyo Kasei Co., Kanto Chemical Co., Tokyo, Japan or Sigma-Aldrich. All chemicals were used as received. Polyviologen **1** was synthesized according to our previous report [3].

### 2.2. Synthesis of Dimethyl-Substituted Polyviologen **2**

Bipyridine precursor was synthesized according to the reported procedure [29]. A mixture of bipyridine (1.5 g, 8.0 mmol) and tetraethylene glycol di(*p*-toluenesulfonate) (4.0 g, 8.0 mmol) was polymerized by stirring at 60 °C for 25 min, 110 °C for 70 min, and 120 °C for 120 min. The product was extracted by chloroform, washed by water, and reprecipitated to acetone several times. Finally, counter ion was exchanged by pouring the polymer solution into an excess amount of LiTFSI aqueous solution [3] to obtain **2** as a brown viscous solid (92% yield).

^1^H NMR (500 MHz; DMSO-d_6_): δ 9.24 (s, 2H, Ph), 9.07 (d, 2H, Ph), 8.15 (d, 2H, Ph), 4.84 (br, 4H, N^+^–CH_2_–), 4.00 (t, 4H, –O–CH_2_–), 3.62 (t, 4H, –O–CH_2_–), 3.52 (t, 4H, –O–CH_2_–), 2.21 (s, 6H, CH_3_). *M*_n_ = 3900. *M*_w_/*M*_n_ = 1.3. Molecular weight was measured by size-exclusion chromatography (SEC, TOSOH HLC-8220) using methanol/acetic acid = 7/3 (*vol*/*vol*) containing 0.5 M sodium acetate as eluent. Polyethylene oxide was used as standard.

### 2.3. Measurements

All measurements were conducted at room temperature. Thin layer polymer electrodes and capacitive counter electrodes covered with ATO (antimony tin oxide) particles were prepared via spin-coating according to our previous procedure [3]. A platinum wire and Ag/AgCl wire were used as counter and reference electrodes, respectively. Before measurements, electrolyte solutions were degassed by nitrogen carefully to remove oxygen. A solution of 1 M LiTFSI aqueous solution was used as electrolyte solution for cyclic voltammetry and chronoamperometry. Cottrell plots were used to determine the diffusion coefficients for charge transfer in polymers [8,34]. For AC impedance, capacitive semicircles of the obtained plots were analyzed by Randles circuits with constant phase elements to evaluate charge transfer resistance, *R*_ct_. Equation (1) was used to estimate exchange current density, *i*_0_ [35].

1/*R*_ct_ = (*i*_0_*AnF*)/(*RT*) (1)

where, *A*: electrode area, *n*: number of electrons for reaction, *F*: Faraday constant, *R*: gas constant, and *T*: temperature.

Electrochemical cells were fabricated by sandwiching polymer and capacitive counter electrodes. Conventional hot melt spacers were used as sealing. Cell gap was approximately 50 μm. Organic electrolytes with wider potential windows (typically > 3 V [36,37]) were used for electrochromic cells. For electrochemical analyses, PGSTAT 128N (AUTOLAB) was employed as a potentiostat. UV-Vis measurements were conducted by USB4000-UV-VIS Ocean Optics. Potential of −2.5 or 2.5 V vs. counter electrode was applied for coloring or decoloring reactions, respectively. The electrochromic cells were photographed after applying −2.5 V for 30 s. Comb-shaped electrodes were used to evaluate ionic conductivity of electrolytes. Viscosity of electrolytes was measured by a vibro-viscometer (SV-1A A&D Corp., San Jose, CA, USA).

Gaussian09 was employed for density functional theory (DFT) calculation. Molecular structures in vacuum were calculated at the B3LYP level of theory with 6-31G(d,p) basis set.

## 3. Results and Discussion

### 3.1. Synthesis of Dimethyl-Substituted Polyviologen and Electrochemical Properties

**Scheme 2:**
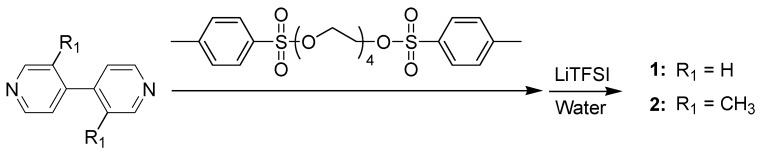
Synthesis of polyviologen **1** and **2**.

As electrochromic polymers, we synthesized polyviologen **1** and **2** with polyethylene glycol backbones by Menschutkin reaction in high yield (>90%, Scheme 2). Polyviologen **1** and **2** consist of normal and dimethyl-substituted bipyridine units as redox centers, respectively. We have already reported **1** as an electrochromic material which was transparent in a dication state and reddish purple in a monocation state [3]. On the other hand, synthesis of polyviologen having substituent on bipyridine groups, such as polymer **2**, seems to be the first attempt so far, and its electrochromic characteristics have not yet been clarified.

**Figure 2:**
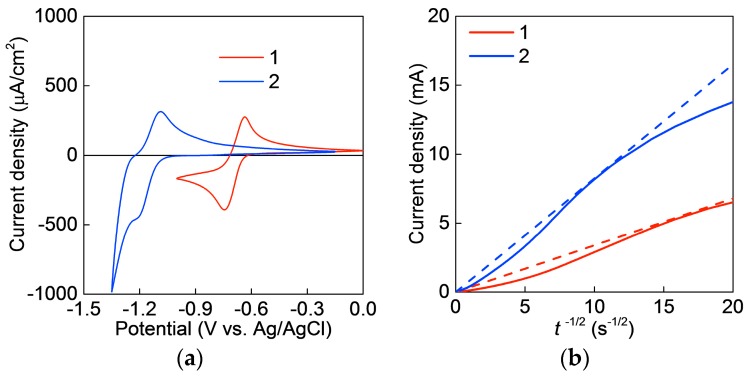
(**a**) Cyclic voltammograms for thin layers of **1** and **2**. Scan rate was 50 mV/s; (**b**) Cottrell curves for **1** and **2**. A potential pulse from −1.2 to 0 V vs. Ag/AgCl was applied. 1 M LiTFSI aqueous solution was used as electrolytes.

To elucidate the electrochemical responses of **1** and **2**, both polymers were spin-coated on ITO substrates and then measured by cyclic voltammetry. The electrodes showed reversible redox waves at *E*_1/2_ = −0.69 V (**1**) and −1.2 V (**2**) vs. Ag/AgCl, corresponding to the redox between the cation and dication states of the bipyridine units (Figure 2a). Dimethyl-substituted polyviologen **2** gave 0.5 V lower redox potential compared to **1**. The difference in the geometry of bipyridines in the cation radical states must have contributed to the potential shift as discussed in the next section. Charge transfer processes via electron self-exchange reaction in polyviologen layers were analyzed by potential step chronoamperometry (Figure 2b) [8,34]. The estimated diffusion coefficients, *D*, for charge transport were 0.24 × 10^−10^ and 1.8 × 10^−10^ cm^2^/s, for **1** and **2**, respectively. The constants were comparable to the previously reported polyviologens (10^−10^ cm^2^/s) [8]. The different molecular geometry of **1** and **2** must have contributed to the difference in diffusion coefficient.

**Figure 3:**
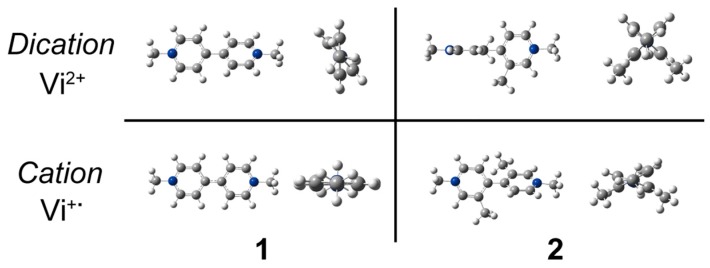
Estimated molecular structures for **1** and **2** in dication and cation states of bipyridine units. Density functional theory (DFT) calculation was used for structure optimization.

Next, we analyzed the molecular structures of polymer **1** and **2** using DFT calculation to estimate their charge transport properties. For simplicity, the methyl viologen structures in vacuum were calculated (Figure 3). In the dication states of **1** and **2**, the pyridine rings twisted more than 45° because of the steric interaction [38]. On the other hand, completely different molecular geometry was observed between the radical cation states of **1** and **2**. Unsubstituted bipyridine **1** gave a structure in which two pyridine rings are arranged in a plane due to the increase in π-conjugation and the tendency to form the quinoid structure [38]. The planar geometry of **1** induced strong stacking between the bipyridines (π-dimerization) [38,39,40] and must have reduced the mobility of the solvent molecules and compensating ions among polymer chains. In contrast, the methyl-substituted pyridine rings **2** were still twisted by 60 degrees because of the strong steric hindrance by methyl groups, which inhibited the quinoid structure and π-stacking of the redox units. Such contrasting electrostatic interaction between the bipyridine rings contributed to the difference in charge transport properties of **1** and **2**. Further, the hindrance in polymer **2** must have reduced the heat of formation of the cation species and thus shifted the redox potential to a lower value.

**Figure 4:**
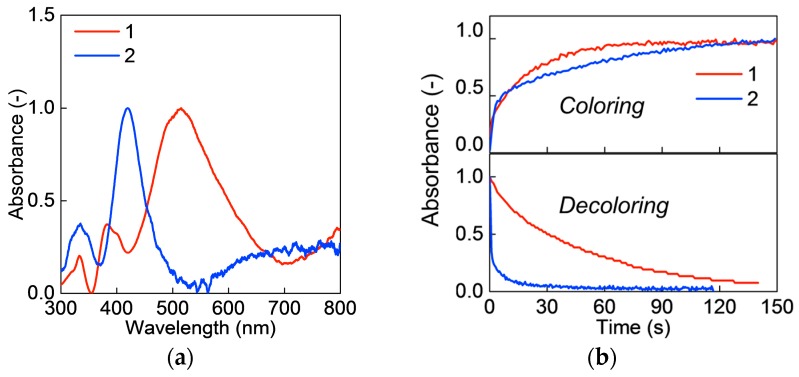
(**a**) UV-Vis spectra for the viologen polymers with cation states. (**b**) Response rate for coloring/decoloring (absorbance at 520 and 420 nm for **1** and **2**, respectively). Electrolyte solution was 35 mM EMIM TCB in 5CB.

The conformation difference of the pyridine rings in **1** and **2** also had significant influences on the electrochromic properties. We prepared electrochromic cells by sandwiching thin layer electrodes of polyviologen and electric double-layer electrodes as counters. The polyviologens gave reversible coloring and decoloring by applying −2.5 and 2.5 V, respectively (Figure 4). Both polymers **1** and **2** were transparent with dication states (2.5 V) but showed purple and blue in cation states (−2.5 V), respectively. The twisting of pyridine rings in **2** definitely shifted the absorption wavelength. Almost the same coloring time was obtained for **1** and **2**, which was comparable to previously reported polyviologen-based electrochromic cells [9]. On the other hand, the decoloring time of **2** (9 s) was 13 times faster than that of **1** (116 s). The excessively strong π-stacking between the bipyridine units in **1** must have suppressed the penetration of counter ions into polymers and thus reduced the decoloring rate as an electrochromic cell. To our knowledge, this is the first report so far of tuning of electrochromic properties of polyviologens by controlling the intermolecular stack. The approach is widely applicable to optimizing absorption wavelength, response rate, and driving voltage for EC polymers.

### 3.2. Suppression of Smudging for Electrochromic Cells

**Figure 5:**
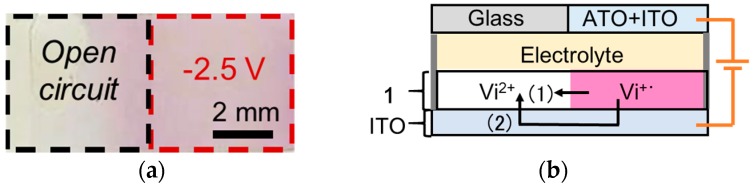
(**a**) Photograph of an electrochromic cell with smudging phenomenon. −2.5 V was applied in a red dashed rectangle area for 30 s. 1 M LiCF_3_SO_3_ in γ-Butyrolactone (GBL) was used as electrolyte solution; (**b**) Structure of an electrochromic device with a partially etched counter electrode.

In order to fabricate electrochromic display with pixels based on the schematic in Figure 1, as a preliminary step, we evaluated the cells in which the left half of the counter electrode was etched (Figure 5). In this configuration, the electrochemical reaction could proceed preferentially only in the right half region to which the voltage was applied. Electrical charge of the polymer must not propagate to the left half, open circuit region to achieve high-resolution of the devices. Here, polyviologen **1** was applied as the electrochromic polymer (**2** was also tested in the last section). When 1 M LiCF_3_SO_3_ in γ-butyrolactone (GBL) was applied as one of common electrolyte solutions, the coloring reaction even progressed to the open circuit region after applying the voltage in 30 s. We suspected that bleeding was caused by (1) electron self-exchange in the polymer and/or (2) charge transfer through the ITO substrate (Figure 5b) because they were the main processes for organic-based electrodes [7]. The “fringing effect” of the electric field [41] could not be the main reason for the smudging (Figure S1, see Supplementary Materials for further discussion).

**Figure 6:**
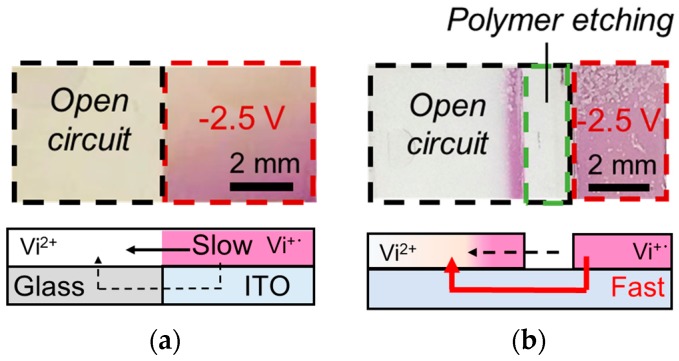
Photographs and structures of electrochromic cells with partially inhibited charge propagation. Charge transport through ITO substrate and polyviologen were inhibited in structure (**a**,**b**), respectively. Electrolyte solution was 1 M LiCF_3_SO_3_ in GBL.

To elucidate the unintended charge transfer processes causing bleeding phenomenon, we prepared the electrochromic devices with the following two special structures (Figure 6). The first was a configuration in which the ITO substrate on the left side of polymer was etched, and the second was in which the polymer region was partially removed. In the former case, smudging was induced only by (1) self-electron exchange reaction in the polymer because the left side of ITO substrate was etched. In the latter case, only (2) charge transfer via ITO contributed to bleeding due to the partial removal of polymer. Bleeding was not observed in the configuration in which ITO was etched (Figure 6a), meaning that the electron self-exchange reaction was not the main reason for smudging. The charge transportable length by the polyviologen, typically expressed by (*Dt*)^0.5^ = 0.5 μm (*t*: time, here *t* = 100 s was used for instance) [7], was sufficiently smaller than the bleed scale (millimeter) and was consistent with the result. On the other hand, in the case where the polymer was partially etched, coloration was observed even in the open circuit region, clearly showing that the smudging was caused by the charge transport via ITO substrate. (Figure 6b).

**Scheme 3:**
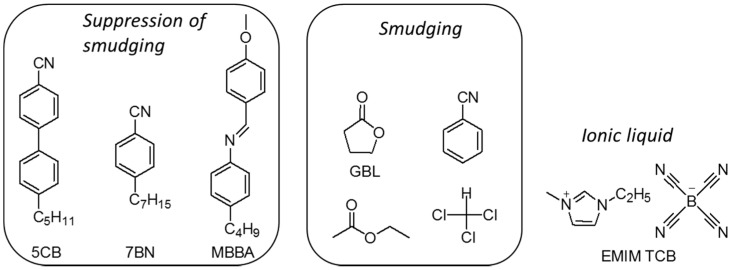
Chemical structures of organic solvent used for smudging test.

**Figure 7:**
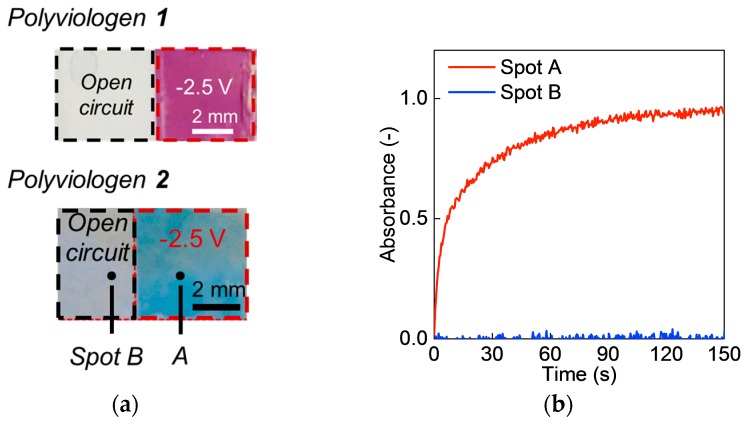
(**a**) Photograph of electrochromic cells containing 35 mM EMIM TCB in 5CB as electrolyte solution. The cell configuration is shown in Figure 5b; (**b**) Time dependence of absorbance at spot A and B (wavelength: 420 nm).

Next, we screened the electrolyte solution which could suppress charge transfer even via ITO, using the normal device configuration shown in Figure 5 (Table 1, Scheme 3). Bleeding was suppressed by applying alkylbenzene compounds containing polar groups such as 4-cyano-4′-pentylbiphenyl (5CB), 4-heptylbenzonitrile (7BN), *N*-(4-methoxybenzylidene)-4-butylaniline (MBBA) as organic solvents with a small amount of ionic liquid, 1-ethyl-1-methylpyrrolidinium tetracyanoborate (EMIM TCB, 35 mM) (Entry 1–3, photograph shown in Figure 7a). 5CB and MBBA have been reported as typical nematic liquid crystals [42] whilst 7BN gave no anisotropy. EMIM TCB was selected as electrolyte salt due to its high miscibility with these solvents [3].

In contrast, bleeding occurred when the salt concentration was too high or when the pristine ionic liquid was used as electrolytes (Entry 4–5). Further, when conventional organic solvents such as GBL and ethyl acetate were used, bleeding progressed regardless of the type and concentration (0 to 1 M) of the electrolyte salts (Entry 6–11). Poorly polar solvents like hexane and toluene were not miscible with EMIM TCB (Entry 12–16). The results meant that the chemical structures of the organic solvents were critical to the suppression of the smudging.

**Figure 8:**
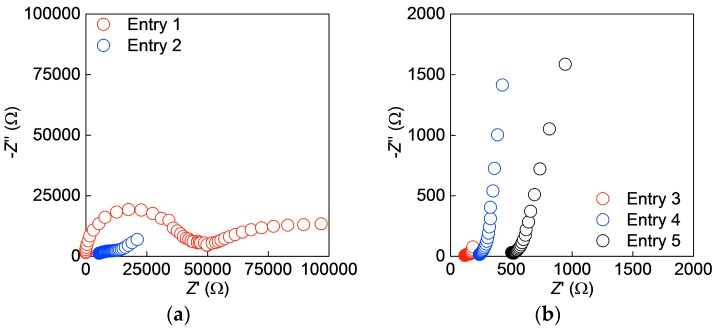
Nyquist plots for thin layer electrodes of **1** measured in a series of electrolytes shown in Table 2. Electrodes were measured at a bias of *E* = *E*_1/2_ vs. Ag/AgCl. AC amplitude was 20 mV (Entry 1–2) or 5 mV (Entry 3–5). Frequency range: ca. 10^4^–10^0^ Hz.

For the elucidation of the factors contributing to the suppression of charge transfer through ITO, the ionic conductivity and solvent viscosity of the electrolytes, and the exchange current density *i*_0_ of the EC electrodes were analyzed (Table 2). The representative cases in Table 1 were tested. Exchange current density was estimated by analyzing charge transfer resistance during AC impedance measurements (Figure 8). Ionic conductivity and viscosity showed no clear relationship with the smudging phenomena although the two factors normally affect the rate of electrochemical reaction [34,43]. In contrast, significantly smaller exchange current density of 10^0^ μA was obtained in the case of alkylbenzene compounds, which suppressed the smudging (Entry 1–2). The current was 10^3^ times smaller than the other conditions. Since charge transfer to the open circuit region proceeded through the ITO substrate, small *i*_0_ must be necessary for the realization of pixels. We note that fast response of the electrochromic devices could also be achieved even with the electrolytes of small *i*_0_ as demonstrated by the fast decoloring of polyviologen **2** (<10 s, Figure 4b, Entry 1). Elucidation of the factors giving small *i*_0_ and fast response of the electrodes, taking into account the condition of inner Helmholtz layer and anisotropy of the field [3,44], are of future interest to us.

The alkylbenzene electrolytes were effective for controlling charge transfer in electrochromic cells regardless of the type of polyviologen **1** or **2** (Figure 7). High optical contrast and moderate cyclability (>10) were observed for EC devices (Figure S2). To quantify the suppression effect of smudging, time evolution of coloring at spot A and B in Figure 7a was measured. Spot A was included in the region where the voltage was applied, and B was located ca. 1 mm away from the voltage application part. Increase in absorbance was observed at spot A, but no change was observed at B even after 150 s of the voltage apply. The resolution was even maintained after the power supply had been stopped (>4 h, Figure S3). The results suggested that the electrochromic displays with high resolution of pixels (at least <1 mm) could be easily fabricated by applying electrolytes with small *i*_0_ and TFT substrates as counter electrodes.

## 4. Conclusions

We synthesized polyviologen containing methyl groups in bipyridine rings for the first time. DFT calculation showed that the twist angle of bipyridine in the radical cation state was increased by the methyl groups and that such strain inhibited the π-interaction between the viologen units. Such conformation change contributed to the shift of the absorption color from ordinary purple to blue and 13-fold enhancement of response rate. Unintended smudging was successfully suppressed by controlling the charge transfer throughout the polymer electrode even if an unconventional, simple device configuration shown in Figure 1b was applied, where a TFT functioned as the counter electrode. Use of alkylbenzene solvents as electrolytes reduced the exchange current of the polymer electrode by a factor of 1/1000 compared to those of the normal electrolytes, inhibiting the unfavorable charge transfer via the ITO substrate. Our findings can be versatilely used for synthesizing multicolor polyviologens with enhanced performances and the fabrication of EC displays with 10^2^–10^3^ μm pixels.

## Figures and Tables

**Table 1:** Organic electrolytes used for smudging tests.

Entry	Solvent	Electrolyte salt	Concentration (mM)	Suppression of smudging
1	5CB	EMIM TCB	35	Yes
2	7BN	EMIM TCB	35	Yes
3	MBBA	EMIM TCB	35	Yes
4	5CB	EMIM TCB	350	No
5	-	EMIM TCB	-	No
6	GBL	EMIM TCB	35	No
7	GBL	LiCF_3_SO_3_	1000	No
8	GBL	LiCF_3_SO_3_	10	No
9	GBL	-	0	No
10	Ethyl acetate	EMIM TCB	35	No
11	Benzonitrile	EMIM TCB	35	No
12	Hexane	EMIM TCB	Immiscible	-
13	Toluene	EMIM TCB	Immiscible	-
14	Chlorobenzene	EMIM TCB	Immiscible	-
15	Dichlorobenzene	EMIM TCB	Immiscible	-
16	4-Pentylbiphenyl	EMIM TCB	Immiscible	-

**Table 2:** Electrochemical parameters for electrolytes solution and thin layer electrodes of **1**.

Entry	Solvent	Electrolyte salt	Concentration (mM)	Ionic conductivity (mS/cm)	Viscosity (mPa∙s) ^1^	Exchange current density *i*_0_ (mA/cm^2^)	Suppression of smudging
1	5CB	EMIM TCB	35	0.012	25 [42]	0.00083	Yes
2	7BN	EMIM TCB	35	0.41	6	0.0074	Yes
3	-	EMIM TCB	-	5.50	23 ^2^	2.1	No
4	GBL	LiCF_3_SO_3_	1000	5.8	1.7 ^2^	2.3	No
5	GBL	LiCF_3_SO_3_	10	0.95	1.7 ^2^	1.4	No
6	GBL	-	0	-	1.7 ^2^	-	No

^1^ Viscosity without electrolyte salts; ^2^ Nominal value by suppliers.

## Supplementary Materials

The following are available online at www.mdpi.com/2073-4360/9/3/86/s1, Figure S1: Heat map of the relative electric field intensity in the partially etched electrochromic cell, Figure S2: (**a**) Reflectance and (**b**) cycle performance of an electrochromic device consisting of polyviologen **1**. (**c**) Reflectance and (**b**) cyclability of **2**. 35 mM EMIM TCB in 5CB was used as an electrolyte. Measured absorbance was at 520 and 420 nm for **1** and **2**, respectively, Figure S3: (**a**) Photographs of an electrochromic cell after coloring reaction. The cell was left at open circuit. The counter electrode was partially etched (configuration shown in Figure 5b). (**b**) Absorbance (at 520 nm) change with time revolution at open circuit. The cell consisted of **1** as an EC layer and 35 mM EMIM TCB in 5CB as an electrolyte.
