# Supplementary material for: Synthesis of Dimethyl-Substituted Polyviologen and Control of Charge Transport in Electrodes for High-Resolution Electrochromic Displays
Source: Polymers (Basel). 2017 Mar 3;9(3):86. doi: 10.3390/polym9030086 (PMC6432454; doi:10.3390/polym9030086)
Supplement: Supplementary file 1 [file polymers-09-00086-s001.pdf]

Supplementary Materials: Synthesis of Dimethyl-Substituted Polyviologen and Control of Charge Transport in Electrode for High-resolution Electrochromic Displays

Kan Sato, Ryusuke Mizukami, Takahiro Mizuma, Hiroyuki Nishide, and Kenichi Oyaizu

To further understand the fringing effect, the static field distribution in an electrochromic cell with a partially etched counter electrode (cell structure shown in Figure 5(b)) was calculated (Figure S1). For simplicity, ITO layers and the electrolyte region were assumed to be metallic and dielectric, respectively. The existence of polymer and ATO layers were ignored. A conventional simulation program (LISA 8.0.0, Sonnenhoff) was employed for the finite element analysis of the static field. The potential of 0 and -2.5 V was applied for the counter and electrochromic sides, respectively. The anisotropy of the liquid crystal electrolytes (5CB and MBBA) could be ignored for the static field calculation (i.e. may not play an important role to explain the experimental results) because smudging was suppressed by even using an isotropic organic solvent (7BN) as an electrolyte.

On the facing region of the etched ITO substrate (part A in Figure S1), a nearly uniform relative electric field intensity $|E| \sim 20$ was observed in a similar way to plate capacitors. The slightly smaller intensity ($|E| = 5–15$) was also observed in the vicinity of part A due to the so-called "fringing effect" [1] ($0 < x < 70$ µm, part B) whilst the intensity reached almost zero at $x > 70$ µm (part C).

The calculation suggested that the "fringing effect" of the static field could induce the pixel smudging on the order of tens of micrometers in the device, but could not be the main reason for the experimentally observed smudging on the several millimeter order.

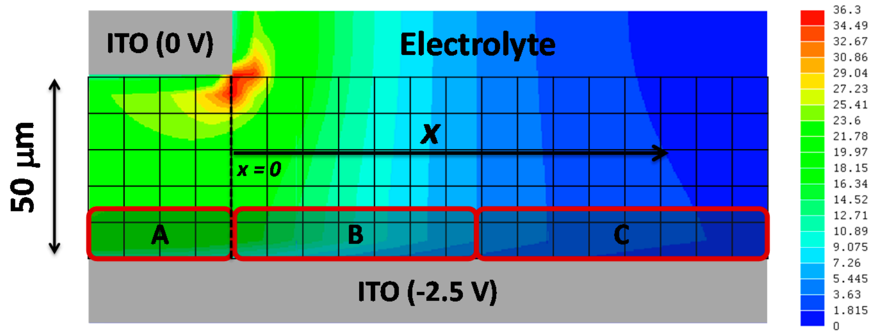

Figure S1. Heat map of the relative electric field intensity in the partially etched electrochromic cell.

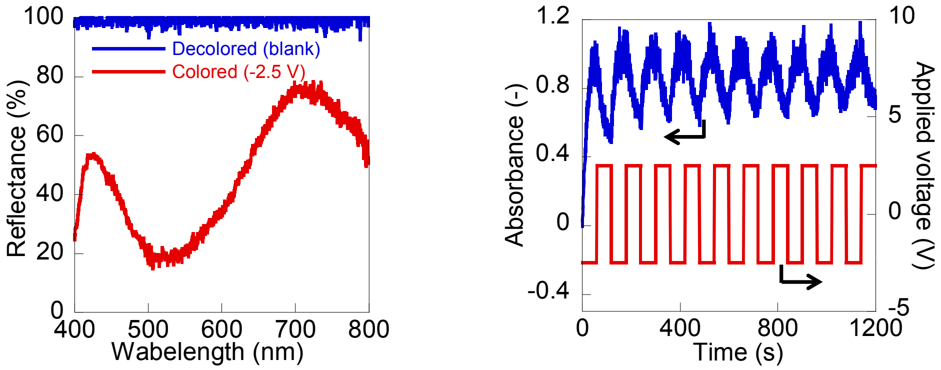

(a) (b)

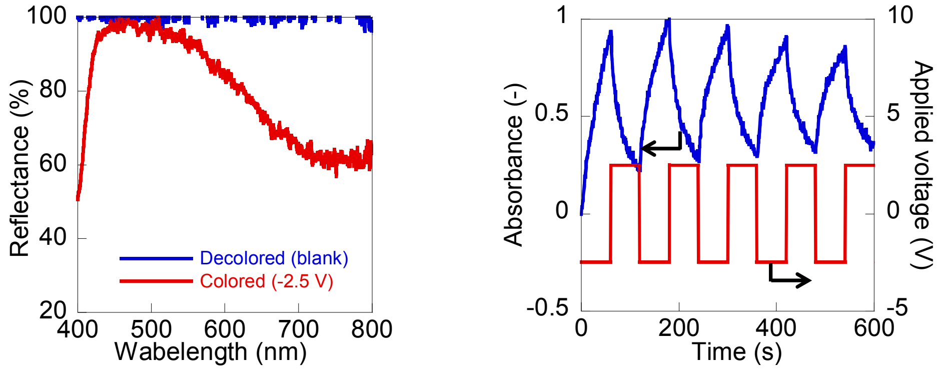

(c) (d)

Figure S2. (**a**) Reflectance and (**b**) cycle performance of an electrochromic device consisting of polyviologen **1**. (**c**) Reflectance and (**b**) cyclability of **2**. 35 mM EMIM TCB in 5CB was used as an electrolyte. Measured absorbance was at 520 and 420 nm for **1** and **2**, respectively.

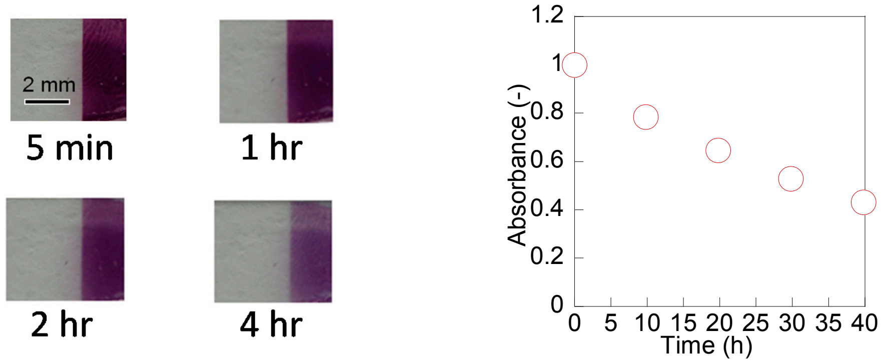

(a) (b)

Figure S3. (**a**) Photographs of an electrochromic cell after coloring reaction. The cell was left at open circuit. The counter electrode was partially etched (configuration shown in Figure 5(b)). (**b**) Absorbance (at 520 nm) change with time revolution at open circuit. The cell consisted of **1** as an EC layer and 35 mM EMIM TCB in 5CB as an electrolyte.

References

1. Lisowski, M.; Skopec, A. Effective area of thin guarded electrode in determining of permittivity and volume resistivity. *IEEE Trans. Dielectr. Electr. Insul.* **2009**, *16*, 24–31.
